# The Heavy Vehicle Study: a case-control study investigating risk factors for crash in long distance heavy vehicle drivers in Australia

**DOI:** 10.1186/1471-2458-10-162

**Published:** 2010-03-26

**Authors:** Mark Stevenson, Lisa N Sharwood, Keith Wong, Jane Elkington, Lynn Meuleners, Rebecca Q Ivers, Ron R Grunstein, Ann Williamson, Narelle Haworth, Robyn Norton

**Affiliations:** 1 The George Institute, The University of Sydney, Sydney Australia; 2 The Woolcock Institute of Medical Research, Sydney, Australia; 3 Curtin University of Technology, Perth, Australia; 4 University of New South Wales, Sydney, Australia; 5 Queensland University of Technology, Brisbane, Australia

## Abstract

**Background:**

Heavy vehicle transportation continues to grow internationally; yet crash rates are high, and the risk of injury and death extends to all road users. The work environment for the heavy vehicle driver poses many challenges; conditions such as scheduling and payment are proposed risk factors for crash, yet the precise measure of these needs quantifying. Other risk factors such as sleep disorders including obstructive sleep apnoea have been shown to increase crash risk in motor vehicle drivers however the risk of heavy vehicle crash from this and related health conditions needs detailed investigation.

**Methods and Design:**

The proposed case control study will recruit 1034 long distance heavy vehicle drivers: 517 who have crashed and 517 who have not. All participants will be interviewed at length, regarding their driving and crash history, typical workloads, scheduling and payment, trip history over several days, sleep patterns, health, and substance use. All participants will have administered a nasal flow monitor for the detection of obstructive sleep apnoea.

**Discussion:**

Significant attention has been paid to the enforcement of legislation aiming to deter problems such as excess loading, speeding and substance use; however, there is inconclusive evidence as to the direction and strength of associations of many other postulated risk factors for heavy vehicle crashes. The influence of factors such as remuneration and scheduling on crash risk is unclear; so too the association between sleep apnoea and the risk of heavy vehicle driver crash. Contributory factors such as sleep quality and quantity, body mass and health status will be investigated. Quantifying the measure of effect of these factors on the heavy vehicle driver will inform policy development that aims toward safer driving practices and reduction in heavy vehicle crash; protecting the lives of many on the road network.

## Background

There is a significant growth in heavy vehicle transportation across the road network not only in high-income countries [1] but also in many developing and newly industrialised countries as a consequence of the rapid, export oriented economic growth [2]. China for example, is observing an annual increase of 466,000 heavy vehicles on their roads [3]. Already the contribution of heavy vehicle crashes to road-related deaths is substantial. In Australia for example, heavy vehicle crashes are responsible for as much as 20% of the total road-related deaths [1,4] whilst in the United States, approximately 15% of road related fatal crashes involve heavy vehicles [5]. Similar rates are reported in the European Union [6]. With high crash rates and an increasing number of heavy vehicles across the road network, there is some urgency to better understand the risks associated with this occupation.

For the heavy vehicle driver, the work environment poses many challenges; vast distances, fixed delivery schedules, low, unregulated freight rates, penalties and carriage of perishable or dangerous goods are some of these. It is proposed that extended driving schedules and inflexible deadlines may contribute to a variety of factors affecting driving behaviour, including fatigue, stress and the use of stimulants in order to stay awake; concerns seen both in Australia and internationally [7-9].

To date, there is inconclusive evidence as to the direction and strength of associations of many postulated risk factors for heavy vehicle crashes including those relating to driver characteristics (including sleep-related factors such as sleep disorders and drowsiness, and health status), vehicle and road environment characteristics as well as employer/company characteristics (including scheduling, shift length and payment). Research in the United States by the Federal Motor Carrier Safety Administration (FMSCA) found that higher remuneration resulted in fewer crashes [10] and Quinlan and Wright [8] recently highlighted a link between heavy vehicle driver remuneration and the risk of crash; however this potential association has not been empirically measured.

Sleep disorders such as obstructive sleep apnoea (OSA) have also been shown to increase the crash risk of motor vehicle drivers by two to seven fold [11]; and this may be higher in the heavy vehicle driver who is over represented in sleep disorder studies. Australian reports have determined 20-30% of heavy vehicle crashes to be sleep related [12]. Sufferers of OSA often experience daytime sleepiness, challenging the performance of tasks requiring vigilance and alertness such as motor vehicle operation [13]. The precise measure of crash risk related to a diagnosis of sleep apnoea, or other measures of daytime sleepiness and driver sleep health, has yet to be quantified.

Although heavy vehicle research has been undertaken, previous studies have significant limitations in part, due to inadequate study design [14] small sample sizes [15,16], the reliance on self-reported crashes and the lack of adjustment for confounders in observational studies [17]. Consequently, there is a need to identify the array of factors contributing to this burgeoning incidence of heavy vehicle crashes and to develop strategies to reduce such. The aim of the proposed study is to determine the direction and magnitude of key postulated risk factors for heavy vehicle crashes, namely:

1. Employer/company related factors such as scheduling, shift length, driving and rest habits of the driver and payment rates and income;

2. Driver characteristics such as fatigue, sleep apnoea, drug and alcohol use and health status including body weight, body mass index and self reported co-morbidities;

3. Vehicle characteristics such as truck configuration and safety features, loads carried and vehicle mass.

## Methods and Design

A population-based case-control study is proposed. The study base includes all drivers of heavy vehicles currently in operation in 3 Australian states namely, New South Wales (NSW), Western Australia (WA) and Queensland (QLD) over a 3-year study period. Drivers will be included if they are undertaking long distance trips (defined as trips in excess of 200 km from their work base), and driving a vehicle ≥12 tonnes. Whilst buses fit this weight criterion, they are excluded from participation in the study due to the significant difference in their scheduling and travel patterns compared to those drivers under study.

### Case Recruitment

Drivers of heavy vehicles that are involved in a crash in one of the above-mentioned states during the 3-year study period will be eligible for recruitment into the study. All crashes must be attended by police and therefore, captured in the respective state police databases. Systematic search strategies have been established that will be run weekly by police across these crash databases. The weekly reports will be sent (via security encrypted files) to the research team, who then send study information and letters of invitation to participate in the study to each heavy vehicle driver that meets the study inclusion criteria. Drivers will be excluded where they have been seriously or fatally injured, or where the crash has resulted in the fatal injury of another road user. Serious injury to the driver is defined as that which 'requires hospitalisation for more than a 1 week period', or the heavy vehicle driver was transported 'unconscious' from the scene of the crash. Telephone contact with those drivers receiving the study information will then be undertaken two weeks from the date the letter was sent. Those drivers not wishing to participate in the study are able to decline via a return mail or alternatively, at the point of telephone contact by the research team. For participating drivers, once informed consent is obtained, a 40 minute telephone interview will be conducted.

### Control Recruitment

Heavy vehicle drivers (as per the definition above) who have not been involved in a police-reported crash in the 12 months leading up to the time of interview will be eligible for recruitment into the study. All refuelling station/truck stop sites across each state have been identified, and heavy vehicle travel patterns across the road network in each state have been obtained from the respective state roads jurisdictions. Using these details, a range of refuelling station/truck stops have been randomly selected to capture a representation of heavy vehicles travelling across the road network in each state. The number of locations selected per state ranges from 6 to 15 sites.

The interviews will be conducted in blocks of time from 4-8 hour time-periods and across the days of the week to ensure weekly travel patterns of heavy vehicle drivers are captured. Once the heavy vehicle refueling station/truck stop and interview time/day schedule is selected, the interviewers will approach heavy vehicle drivers entering the refueling station/truck stop to participate in the study. Interviewers will continue recruiting at each site until the available or recommended time period has elapsed. Demographic information such as age, height, weight and loads carried will be requested of non-participants.

Both case and control interviews are conducted by a consistent group of staff all trained using a standardized protocol. As well, since the diagnosis of sleep apnoea among the case subjects could be sensitive and may have ramifications for their ongoing employment, we will be emphasising strongly the confidential nature of the study. Given the importance of maintaining a sufficient response fraction, rigorous attention will be paid to recruitment strategies and the development of rapport with potential participants. However, in anticipation of those drivers who decline participation in the study, and to minimise the potential for bias, we will collect demographic data on these drivers. Further, ensuring methodological rigour, we are using validated diagnostic measures for potential risk factors where possible.

### Driver Interview and Sleep Apnoea testing

A questionnaire was developed by consensus of a panel comprising experts in transportation, the heavy vehicle industry, fatigue and sleep disorders, legislation, injury prevention, and road safety. The questionnaire collects information about the driver's characteristics including demographics, anthropometry, health status, drug and alcohol use, sleep quantity and quality and fatigue. For self reported measures of sleepiness, the questionnaire includes standardised, validated questions such as the Epworth Sleepiness Scale [18], and Multivariable Apnoea Prediction Index [19]. Summary information from all drivers will include their driving and crash history, income details, distances travelled and hours worked. Vehicle information obtained will include truck weight and configuration, safety features and typical loads carried. Detailed information will be further collected about work schedules, travel patterns and sleep (quantity and quality) for the three days preceding the crash (for cases) or the three days preceding the face to face interview (for controls).

In order to test for obstructive sleep apnoea, all drivers will be sent a diagnostic device (Flow Wizard™) to wear overnight during their sleep. This device is a validated nasal pressure transducer that non-invasively measures an individual's airflow during sleep and will detect apnoeas, hypopnoeas and snoring per hour of recording [20,21]. It can be worn with minimum instruction and will be mailed back to the research team for analysis. The device has shown reliable accuracy in detecting obstructive sleep apnoea (sensitivity of 0.92), and with sufficient data can classify results as 'no/minimal', 'moderate' or 'severe' disease [21].

### Study Power

A total sample of 1034 heavy vehicle drivers will be recruited comprising 517 cases and 517 controls. A number of studies have shown that the risk of crashing for heavy vehicle drivers doubles with increased hours of driving [22]. Drivers in a New Zealand study were 2.6 times more likely to crash if they had driven 8 or more hours since their last compulsory 10 hour break, compared with those who had driven less than 8 hours [15]. Similarly, research in the United States reported a 2.2 fold increase in the likelihood of crash for each additional 100 km travelled [23]. With regard to sleep/fatigue related covariates, Santos et al [24] reported an association between a diagnosis of sleep apnoea and the risk of crash among car drivers, and hours of sleep over the previous day have been associated with an increased risk of crash in car drivers (< 5 hrs, OR 2.7) as well as those driving between 2-5 am (0.4%, OR 5.6) [25]. In the absence of research identifying the association between specific schedules/payment and crash, the power for the study was estimated on associations between heavy vehicle crash and sleep related conditions. Therefore taking a conservative prevalence estimate of 5%, with an expected odds ratio of 2.0, the minimum sample size necessary to detect a true odds ratio with 80% power is 517 cases and 517 controls.

### Statistical Analysis

Descriptive statistics will profile the study population on all demographic data and survey responses, and then comparisons will be made between the cases and controls. A series of univariate analyses will be conducted using chi square tests for categorical variables and t tests for continuous variables. Factors that appear to be associated with crash risk will be included in a multivariate model, determining the odds of crash. The odds ratio will be estimated related to the key aims of the study namely, factors about payment, hours worked, shift scheduling and a diagnosis of sleep apnoea. Unconditional logistic regression analysis will be undertaken, adjusting for confounding factors. For all tests, probability values of less than .05 will be considered statistically significant. Odds ratios and 95% confidence intervals will be derived using the standard case-control study methods described by Breslow and Day [26].

The control group of 517 drivers will then be analysed separately, (as a prevalence survey) to describe the prevalence of key risk factors among heavy vehicle drivers. Statistical calculations will be performed on STATA software (version 10.0 Stata Corporation, College Station, TX, USA).

### Informed Consent and Ethics

Informed consent will be obtained from all participants; control drivers will be read a consent statement after provision of a description of the study, and then will sign the consent form (with a second copy for their records). For case drivers, where interviews are conducted over the telephone, they will be informed about the study, and a supervisor of the interviewer will listen to the consent process on a second telephone line. The supervisor will confirm the consent process in writing.

Ethics approval for the study was been obtained in January 2008 from The University of Sydney Human Research Ethics Committee; prior to approval by Curtin University Human Research Ethics Committee in WA and the Ethical Standing division of Queensland Police in Qld. All applications for amendment with refined versions of Participant Information Statements, Interviews, Consent forms etc. have been approved.

## Discussion

Heavy vehicle crashes contribute significantly to the burden of death and disability seen on our roads. Considerable attention has been paid to the enforcement of legislation aiming to deter problems such as excess loading, speeding and substance use; however, there is inconclusive evidence as to the direction and strength of associations of many other postulated risk factors for heavy vehicle crashes. The management and regulation of heavy vehicles particularly in relation to safety, is recognised as an urgent issue at all levels of government. In the absence of research that identifies the key determinants of heavy vehicle crashes; preventive strategies will continue to be based on anecdote. The proposed study addresses key elements highlighted in the World Health Organisation's World Report on Road Traffic Injury Prevention [27] by determining the role of key risk factors in crash involvement of heavy vehicle drivers. Quantifying the contribution of factors such as scheduling, payment and sleep-related factors in heavy vehicle crashes will enable identification of cost-effective strategies to reduce the associated growing injury burden.

## Competing interests

The authors declare that they have no competing interests.

## Authors' contributions

MS and RI lead the methodological design of the study, supported by RN, LM, RG, AW and NH. LNS and MS drafted the paper and LNS incorporated revisions between authors. All authors read and approved the final manuscript.

## Pre-publication history

The pre-publication history for this paper can be accessed here:

http://www.biomedcentral.com/1471-2458/10/162/prepub
